# Comparison of the risk of gastrointestinal perforation between patients with and without rheumatoid arthritis: A nationwide cohort study in Asia

**DOI:** 10.3389/fmed.2022.974328

**Published:** 2022-09-29

**Authors:** Ting-Chia Chang, Wei-Chih Kan, Kuo-Chen Cheng, Chung-Han Ho, Yi-Chen Chen, Chin-Chen Chu, Chien-Chin Hsu, Hsing-Tao Kuo, Hung-Jung Lin, Chien-Cheng Huang

**Affiliations:** ^1^Department of Internal Medicine, Chi Mei Medical Center, Tainan, Taiwan; ^2^Department of Intensive Care Medicine, Chi Mei Medical Center, Tainan, Taiwan; ^3^Department of Biological Science and Technology, Chung Hwa University of Medical Technology, Tainan, Taiwan; ^4^Department of Medical Research, Chi Mei Medical Center, Tainan, Taiwan; ^5^Department of Information Management, Southern Taiwan University of Science and Technology, Tainan, Taiwan; ^6^Department of Anesthesiology, Chi Mei Medical Center, Tainan, Taiwan; ^7^Department of Emergency Medicine, Chi Mei Medical Center, Tainan, Taiwan; ^8^Department of Emergency Medicine, Taipei Medical University, Taipei, Taiwan; ^9^Department of Emergency Medicine, Kaohsiung Medical University, Kaohsiung, Taiwan; ^10^Department of Environmental and Occupational Health, College of Medicine, National Cheng Kung University, Tainan, Taiwan

**Keywords:** gastrointestinal perforation, rheumatoid, arthritis, cohort study, Taiwan

## Abstract

**Objectives:**

Patients with rheumatoid arthritis (RA) may have an increased risk for gastrointestinal perforation (GIP) caused by medications or chronic inflammation. However, the risk of GIP between patients with and without RA remains unclear. Therefore, we conducted this study to clarify it.

**Methods:**

Using the Taiwan National Health Insurance Research Database, we identified patients with and without RA matched at 1:1 ratio by age, sex, and index date between 2000 and 2013 for this study. Comparison of the risk of GIP between the two cohorts was performed by following up until 2014 using Cox proportional hazard regression analyses.

**Results:**

In total, 11,666 patients with RA and an identical number of patients without RA were identified for this study. The mean age (±standard deviation) and female ratio were 55.3 (±15.2) years and 67.6% in both cohorts. Patients with RA had a trend of increased risk for GIP than patients without RA after adjusting for underlying comorbidities, medications, and monthly income [adjusted hazard ratio (AHR) 1.42; 95% confidence interval (CI) 0.99–2.04, *p* = 0.055]. Stratified analyses showed that the increased risk was significant in the female population (AHR 2.06; 95% CI 1.24–3.42, *p* = 0.005). Older age, malignancy, chronic obstructive pulmonary disease, and alcohol abuse were independent predictors of GIP; however, NSAIDs, systemic steroids, and DMARDs were not.

**Conclusion:**

RA may increase the risk of GIP, particularly in female patients. More attention should be paid in female population and those with independent predictors above for prevention of GIP.

## Introduction

Gastrointestinal perforation (GIP) is a potentially lethal medical condition, which has various causes, including ischemia (e.g., bowel obstruction and necrosis), infection (e.g., appendicitis and diverticulitis), erosion (e.g., malignancy and ulcerative disease), and physical disruption (e.g., trauma, iatrogenic injury, and foreign body) (1, 2). GIP always need surgery unless the patient cannot tolerate it or chooses not to receive it (1). GIP has a mortality rate of up to 30% or higher (3), depending on the patient's age, medical comorbidities, benign or malignant cause, and functional status (1).

Rheumatoid arthritis (RA) is considered a risk factor for GIP because of RA medications, including non-steroidal anti-inflammatory drugs (NSAIDs), steroids, and disease-modifying antirheumatic drugs (DMARDs) (4–6). The inflammation or other processes in RA may be also a risk factor for GIP (4, 7). Many studies have reported the comparison of the risk of GIP using medications, including biologic agents, DMARDs, steroids, and NSAIDs (4, 5, 7). Some studies have reported risk factors for GIP in patients with RA (5, 8). However, the comparison of the risk of GIP between patients with and without RA remains unclear. Most studies about GIP in RA are also conducted in Western countries, and investigations in Asian countries are limited. Therefore, we conducted this study to fill the data gap.

## Methods

### Data sources

We conducted this nationwide population-based cohort study using data from Taiwan's National Health Insurance Research Database (NHIRD). Taiwan NHIRD is based on the Taiwan National Health Insurance program, which is a compulsory social insurance system covering nearly all Taiwanese population (9). The National Health Insurance program consists of registration files and anonymous claims data for reimbursement (9). The accuracy of the Taiwan NHIRD has been validated in many studies (9–11).

### Study design, setting, and participants

We identified patients with RA from the Taiwan NHIRD between 2000 and 2013 as the study cohort. The criteria of RA were the diagnosis of RA [International Classification of Diseases, Ninth Revision, Clinical Modification (ICD-9-CM) code 714] in at least three outpatient visits or one hospitalization (12). Patients who were diagnosed with GIP (ICD-9-CM codes 530.4, 531.1, 531.2, 531.5, 531.6, 532.1, 532.2, 532.5, 532.6, 533.1, 533.2, 533.5, 533.6, 534.1, 534.2, 534.5, 534.6, and 569.83, 540.0) before the diagnosis of RA were excluded. The criteria of GIP should also be made in at least three outpatient visits or one hospitalization. We identified patients without RA as the comparison cohort by matching them with patients with RA in a 1:1 ratio using age, sex, and index date. The index date was the date that the patient with RA was diagnosed with RA. The comparison cohort also excluded patients with GIP before the index date.

### Definitions of variables

Age was classified as the following subgroups: <35, 35–44, 45–54, 55–64, 65–74, 75–84, and ≥85 years (12). Underlying comorbidities analyzed were diseases of the esophagus, stomach, and duodenum (ICD-9-CM codes 530–539), hypertension (ICD-9-CM codes 401–405), mental disorder (ICD-9-CM codes 290–319), liver disease (ICD-9-CM codes 570–576), hyperlipidemia (ICD-9-CM code 272), renal disease (ICD-9-CM codes 580–593), coronary artery disease (ICD-9-CM codes 410–414), diabetes (ICD-9-CM code 250), stroke (ICD-9-CM codes 436–438), malignancy (ICD-9-CM codes 140–208), chronic obstructive pulmonary disease (ICD-9-CM code 496), alcohol abuse (ICD-9-CM code 291, 303, 3050, 3575, 4255, 5353, 5710-5713, and V113), and diverticula of the intestine (ICD-9-CM code 562). These underlying comorbidities should be present in at least three outpatient visits or one hospitalization before the index date. There was no time-window for the underlying comorbidities. The medications used were NSAIDs [Anatomical Therapeutic Chemical (ATC) codes M01AB, M01AC, M01AE, M01AG, and M01AH], systemic steroids (ATC code H02AB), conventional DMARDs [ATC codes L04AX03 (oral form of Methotrexate), L01BA01 (injection form of Methotrexate), L04AA13 (oral form of Leflunomide), A07EC01 (oral form of Sulfasalazine), L04AD01 (oral form of Cyclosporine), P01BA02 (oral form of Hydroxychloroquine), L04AX01 (oral form of Azathioprine)], and biologic DMARDs [ATC codes L04AB01 (injection form of Etanercept), L04AB06 (injection form of Golimumab), L01FA01 (injection form of Rituximab), and L04AC07 (injection form of Tocilizumab)] for at least 7 days. Taiwan National Health Insurance is a single-payer system, which include nearly all the medications of the citizens in Taiwan. Therefore, we captured the information about medications from the NHIRD only. The medication information came from the data in the NHIRD, not from the report by the patients. Monthly income was classified in three levels: <20,000, 20,000–40,000, and ≥40,000 New Taiwan Dollars (NTD) (12).

### Outcome measurements

The two cohorts were followed until 2014 and compared for the development of GIP. We further divided GIP into two subgroups for the outcome analyses, including upper GIP and lower GIP. Upper GIP was defined by ICD-9-CM codes (esophagus 530.4, and stomach 531.1, 531.2, 531.5, 531.6, 532.1, 532.2, 532.5, 532.6, 533.1, 533.2, 533.5, and 533.6), and lower GIP was defined by ICD-9-CM codes (small intestine 534.1, 534.2, 534.5, 534.6 and large intestine 540.0, 569.83) according to the previous study about GIP in RA patients (13). In addition, all patients were right censored on the date of death, lost to follow-up, or end date of study period, December 31, 2014.

### Ethics statement

The study was conducted in compliance with the ethical principles of the Helsinki Declaration and approved by the Institutional Review Board of Chi Mei Medical Center. Informed consent was waived because of the anonymous use of data with subjects unidentifiable before analysis.

### Statistical analysis

We used Pearson chi-square test and independent *t*-test to analyze categorical and continuous variables between the two cohorts. Considering of the time until events occur in cohort study, univariate and multivariate Cox proportional hazards regression analyses were performed to estimate the risk of GIP between RA cohort and comparison cohort. Stratified analyses were performed according to upper GIP, lower GIP, age, sex, underlying comorbidities, medications, monthly income, and follow-up periods to investigate potential effect modification. We also performed Cox proportional hazard regression analyses in all patients to investigate independent predictors for GIP. The Kaplan-Meier analysis was performed to compare the cumulative incidence of GIP between the two cohorts. All statistical analyses were performed using SAS 9.4 for Windows (SAS Institute, Cary, NC, USA). A *p*-value <0.05 indicated significance (two-tailed).

## Results

For this study, we identified a total of 11,666 patients with RA and 11,666 patients without RA (Table 1). The mean age (standard deviation) in both cohorts were 55.3 (15.2) years, with a predominance of female patients (67.6%). The proportion of patients aged 45–54 years was the highest (25.3%), followed by those aged 55–64 (22.7%), 65–74 (18.0%), and 35–44 (14.0%). No significant differences were found in age and sex between the two cohorts because of matching. Compared with patients without RA, those with RA had a higher prevalence of underlying comorbidities (namely, including diseases of the esophagus, stomach, and duodenum, hypertension, mental disorder, liver disease, hyperlipidemia, renal disease, coronary artery disease, diabetes, stroke, chronic obstructive pulmonary disease) and alcohol abuse and higher use of NSAIDs, systemic steroids, and DMARDs. Patients with RA had a lower monthly income than those without RA. Patients with RA had a higher risk of overall GIP and upper GIP than patients without RA (0.7 vs. 0.5%).

**Table 1:** Comparison of demographic characteristics, underling comorbidities, medications, and monthly income between patients with and without RA.

**Variable**	**With RA**	**Without RA**	***p*** **-value**
	***n*** ** = 11,666**	***n*** ** = 11,666**	
Age (years)	55.3 ± 15.2	55.3 ± 15.2	0.961
**Age subgroups**			
<35	1,042 (8.9)	1,045 (9.0)	0.998
35–44	1,634 (14.0)	1,633 (14.0)	
45–54	2,955 (25.3)	2,953 (25.3)	
55–64	2,647 (22.7)	2,651 (22.7)	
65–74	2,103 (18.0)	2,098 (18.0)	
75–84	1,152 (9.9)	1,154 (9.9)	
≥85	133 (1.1)	132 (1.1)	
**Sex**			
Female	7,887 (67.6)	7,887 (67.6)	>0.999
Male	3,779 (32.4)	3,779 (32.4)	
**Underlying comorbidities**			
Diseases of the esophagus, stomach, and duodenum	7,148 (61.3)	5,547 (47.6)	<0.001
Hypertension	4,233 (36.3)	3,595 (30.8)	<0.001
Mental disorder	4,023 (34.5)	2,770 (23.7)	<0.001
Liver disease	3,036 (26.0)	2,120 (18.2)	<0.001
Hyperlipidemia	2,679 (23.0)	2,067 (17.7)	<0.001
Renal disease	2,065 (17.7)	1,457 (12.5)	<0.001
Coronary artery disease	2,049 (17.6)	1,606 (13.8)	<0.001
Diabetes	1,875 (16.1)	1,642 (14.1)	<0.001
Stroke	733 (6.3)	617 (5.3)	0.001
Malignancy	623 (5.3)	577 (5.0)	0.173
Chronic obstructive pulmonary disease	461 (4.0)	339 (2.9)	<0.001
Alcohol abuse	205 (1.8)	107 (0.9)	<0.001
Diverticula of intestine	62 (0.5)	47 (0.4)	0.150
**Medications**			
NSAIDs	11,516 (98.7)	10,795 (92.5)	<0.001
Systemic steroids	1,113 (9.5)	581 (5.0)	<0.001
DMARDs	1,285 (11.0)	122 (1.1)	<0.001
**Monthly income ($NTD)**			
<20,000	8,900 (76.3)	8,638 (74.0)	<0.001
20,000–40,000	1,929 (16.5)	2,032 (17.4)	
≥40,000	837 (7.2)	996 (8.6)	
Overall GIP	84 (0.7)	56 (0.5)	0.018
**Anatomy classification**			
Upper GIP	50 (0.4)	27 (0.2)	0.009
Lower GIP	35 (0.3)	29 (0.3)	0.453
Time to event (GIP), Median (Q1–Q3)	4.4 (1.8–7.0)	4.4 (1.4–7.2)	

Data were expressed as n (%) or mean ± SD. GIP, gastrointestinal perforation; RA, rheumatoid arthritis; NSAIDs, nonsteroidal anti-inflammatory drugs; DMARDs, disease-modifying antirheumatic drugs; NTD, New Taiwan Dollars.

**Figure 1:**
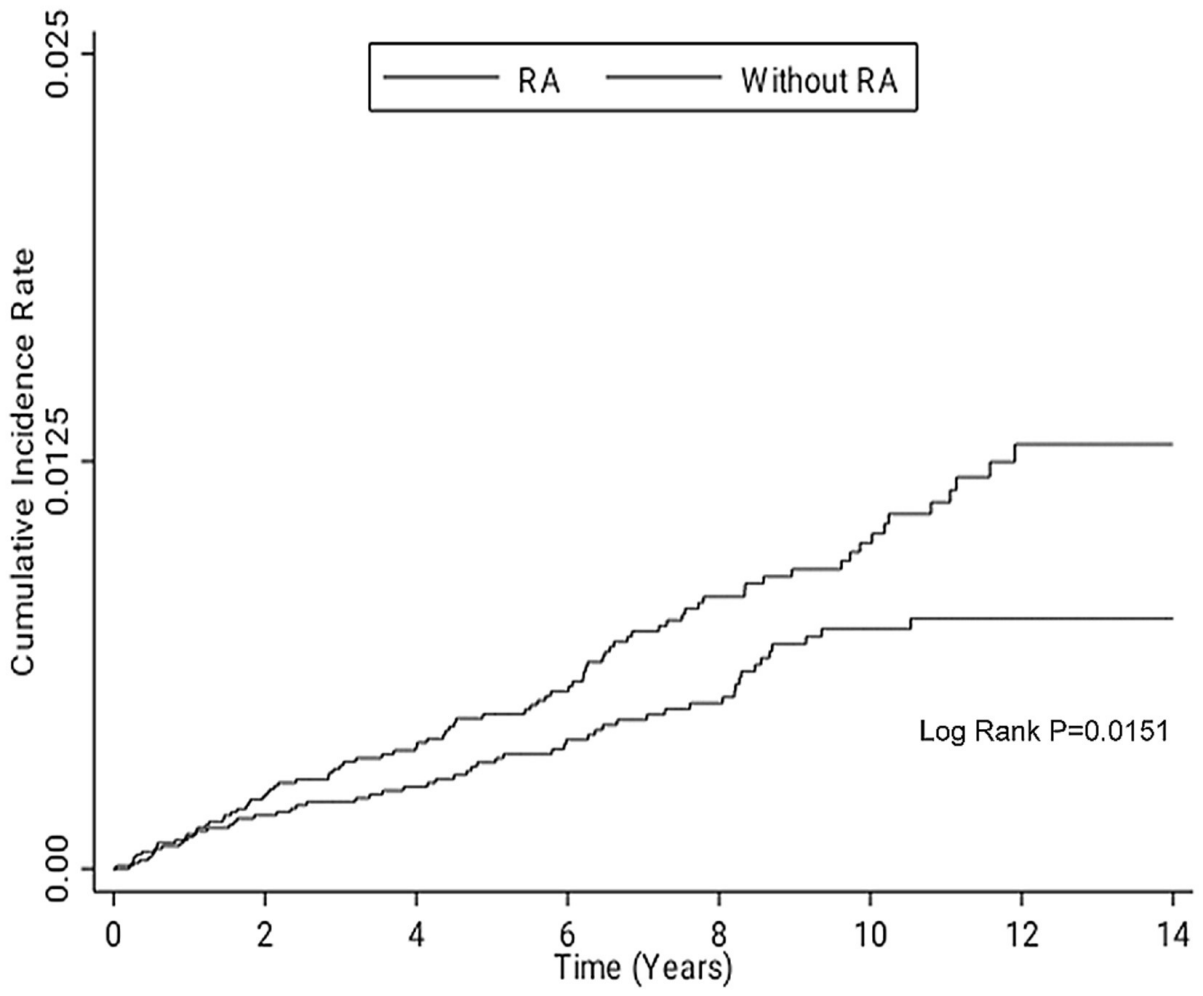
Kaplan–Meier survival curve for comparing the risk of GIP in the patients with and without RA after following up for 14 years. GIP, gastrointestinal perforation; RA, rheumatoid arthritis.

In overall GIP, Cox proportional hazards regression analyses showed patients with RA had an increased crude hazard ratio (HR) (1.52) than patients without RA [95% confidence interval (CI) 1.08–2.13] (Table 2). However, the difference became statistically non-significant after adjusting for age, sex, underlying comorbidities, medications, and monthly income [adjusted hazard ratio (AHR) 1.42; 95% CI 0.99–2.04, *p* = 0.055]. The overall GIP in patients with RA was 1.0/1,000 person-years. In the comparison of the risk of upper GIP, patients with RA had a trend of increased risk than patients without RA (AHR 1.62; 95% CI 0.99–2.66, *p* = 0.055). There was no significant difference of the risk of lower GIP between the two cohorts (AHR 1.24; 95% CI 0.73–2.09, *p* = 0.432). In patients with RA who had GIP, 58.8% (50/85) and 41.2% (35/85) of the cases were upper GIP and lower GIP. Female patients with RA had an increased risk of GIP than female patients without RA (AHR 2.06; 95% CI 1.24–3.42, *p* = 0.005); however, no difference was found in the male population. As regards the duration of follow-up, the period of 6–8 years showed a significant difference of the risk of GIP between the two cohorts. Stratified analyses according to age, underlying comorbidities, medications, and monthly income did not show significant difference between the two cohorts. The Kaplan–Meier survival curve presented an increased risk of GIP in patients with RA than in those without RA after follow-up for 14 years (Figure 1). Older age, malignancy, chronic obstructive pulmonary disease, and alcohol abuse were independent predictors of GIP; however, NSAIDs, systemic steroids, and DMARDs were not (Table 3).

**Table 2:** Comparison of the risk for GIP between patients with and without RA by Cox proportional hazard regression analyses.

**Variable**	**With RA**			**Without RA**			**Crude HR** **(95% CI)**	**AHR (95%** CI)^*^	***p*** **-value^†^**
	**GIP (%)**	**PY**	**rate^@^**	**GIP (%)**	**PY**	**rate^@^**			
**Overall analysis**	85 (0.7)	82,603.9	1.0	56 (0.5)	83,564.9	0.7	1.52 (1.08–2.13)	1.42 (0.99–2.04)	0.055
Upper GIP	50 (0.4)	82,712.9	0.6	27 (0.2)	83,663.3	0.3	1.87 (1.17–2.99)	1.62 (0.99–2.66)	0.055
Lower GIP	35 (0.3)	82,767.3	0.4	29 (0.3)	83,651.7	0.3	1.22 (0.75–1.99)	1.24 (0.73–2.09)	0.432
**Stratified analysis**									
Age (years)									
<35	2 (2.4)	8,416.6	0.2	1 (1.8)	8,481.5	0.1	2.01 (0.18–22.11)	1.84 (0.16–20.76)	0.622
35–45	10 (11.9)	13,012.2	0.8	6 (10.7)	13,180.7	0.5	1.68 (0.61–4.62)	2.15 (0.73–6.38)	0.166
45–55	12 (14.3)	21,338.6	0.6	7 (12.5)	21,257.7	0.3	1.59 (0.62–4.10)	1.56 (0.54–4.54)	0.414
55–65	14 (16.7)	18,428.3	0.8	11 (19.6)	18,505.3	0.6	1.17 (0.54–2.53)	1.18 (0.51–2.76)	0.696
65–75	22 (26.2)	14,396.3	1.5	18 (32.1)	15,065.5	1.2	1.39 (0.74–2.61)	1.30 (0.66–2.54)	0.451
75–85	21 (25.0)	6,511.1	3.2	11 (19.6)	6,546.3	1.7	1.77 (0.87–3.60)	1.39 (0.67–2.91)	0.376
≥85	3 (3.6)	500.8	6.0	2 (3.6)	527.9	3.8	3.10 (0.32–29.85)		
**Sex**									
Female	55 (65.5)	55,361.9	1.0	26 (46.4)	55,701.1	0.5	2.13 (1.33–3.39)	2.06 (1.24–3.42)	0.005
Male	29 (34.5)	27,242.0	1.1	30 (53.6)	27,863.7	1.1	0.99 (0.59–1.64)	0.95 (0.56–1.62)	0.858
**Underlying comorbidity**									
Diseases of the esophagus, stomach, and duodenum	44 (52.4)	44,044.4	1.0	29 (51.8)	32,471.0	0.9	1.12 (0.70–1.80)	1.09 (0.68–1.77)	0.717
Hypertension	41 (48.8)	26,233.4	1.6	25 (44.6)	22,015.4	1.1	1.38 (0.84–2.26)	1.27 (0.76–2.12)	0.372
Mental disorder	32 (38.1)	23,762.9	1.4	19 (33.9)	15,980.9	1.2	1.14 (0.64–2.01)	1.16 (0.64–2.10)	0.619
Liver disease	19 (22.6)	18,066.7	1.1	18 (32.1)	12,217.8	1.5	0.71 (0.37–1.36)	0.72 (0.37–1.39)	0.323
Hyperlipidemia	18 (21.4)	14,803.9	1.2	10 (17.9)	11,315.1	0.9	1.38 (0.64–2.98)	1.22 (0.54–2.75)	0.627
Renal disease	16 (19.1)	11,577.5	1.4	10 (17.9)	8,237.4	1.2	1.13 (0.51–2.50)	0.91 (0.40–2.08)	0.819
Coronary artery disease	16 (19.1)	12,209.1	1.3	12 (21.4)	9,394.1	1.3	1.02 (0.48–2.17)	1.03 (0.48–2.24)	0.936
Diabetes	16 (19.1)	11,164.9	1.4	13 (23.2)	9,610.7	1.4	1.06 (0.51–2.20)	0.92 (0.43–1.98)	0.839
Stroke	7 (8.3)	4,232.8	1.7	8 (14.3)	3,205.5	2.5	0.68 (0.25–1.89)	0.51 (0.17–1.54)	0.235
Malignancy	7 (8.3)	3,153.3	2.2	7 (12.5)	3,056.4	2.3	0.95 (0.33–2.72)	0.68 (0.21–2.24)	0.525
Chronic obstructive pulmonary disease	11 (13.1)	2,475.5	4.4	6 (10.7)	1,798.1	3.3	1.33 (0.49–3.59)	1.48 (0.52–4.22)	0.463
Alcohol abuse	4 (4.8)	1,076.2	3.7	1 (1.8)	528.2	1.9	2.02 (0.23–18.07)	–	–
Diverticula of the intestine	1 (1.2)	303.8	3.3	2 (3.6)	249.2	8.0	0.37 (0.03–4.13)	–	–
**Medications**									
NSAIDs	82 (97.6)	81,234.2	1.0	49 (87.5)	75,030.7	0.7	1.55 (1.09–2.20)	1.40 (0.97–2.02)	0.071
Systemic steroids	10 (11.9)	5,676.5	1.8	2 (3.6)	3,241.6	0.6	2.82 (0.61–12.77)	1.75 (0.34–9.07)	0.505
DMARDs	11 (13.1)	8,155.7	1.3	1 (1.79)	707.2	1.4	0.95 (0.12–7.38)	2.12 (0.17–26.75)	0.561
**Monthly income ($NTD)**									
<20,000	43 (51.2)	27,916.6	1.5	27 (48.2)	28,528.9	1.0	1.61 (1.11–2.33)	1.47 (1.00–2.18)	0.053
20,000–40,000	38 (45.2)	48,682.2	0.8	26 (46.4)	47,752.5	0.5	0.95 (0.34–2.61)	1.39 (0.44–4.42)	0.579
≥40,000	3 (3.6)	6,005.1	0.5	3 (5.4)	7,283.5	0.4	1.22 (0.25–6.04)	1.12 (0.15–8.29)	0.911
**Follow–up period**									
<6 months	7 (8.3)	171.9	40.7	9 (16.1)	160.9	55.9	1.50 (0.42–5.32)	1.45 (0.33–6.33)	0.620
6–12 months	5 (6.0)	265.2	18.9	2 (3.6)	247.8	8.1	0.86 (0.29–2.56)	0.75 (0.24–2.31)	0.612
1–2 years	12 (14.3)	1,325.1	9.1	7 (12.5)	1,323.4	5.3	1.73 (0.68–4.38)	1.66 (0.62–4.43)	0.314
2–4 years	14 (16.7)	4,800.6	2.9	8 (14.3)	4,832.9	1.7	1.77 (0.74–4.21)	1.39 (0.57–3.43)	0.470
4–6 years	13 (15.5)	7,397.8	1.8	11 (19.6)	7,466.1	1.5	1.19 (0.53–2.66)	1.02 (0.42–2.53)	0.959
6–8 years	18 (21.4)	11,349.5	1.6	7 (12.5)	11,130.4	0.6	2.60 (1.09–6.23)	2.51 (1.01–6.27)	0.049
≥8 years	15 (17.9)	57,293.9	0.3	12 (21.4)	58,403.5	0.2	1.28 (0.60–2.73)	1.43 (0.63–3.24)	0.391

GIP, gastrointestinal perforation; RA, rheumatoid arthritis; PY, person-year; CI, confidence interval; AHR, adjusted hazard ratio; NSAIDs, nonsteroidal anti-inflammatory drugs; DMARDs, disease-modifying antirheumatic drugs; NTD, New Taiwan Dollars.

* Adjusted for diseases of the esophagus, stomach, and duodenum, hypertension, mental disorder, liver disease, hyperlipidemia, renal disease, coronary artery disease, diabetes, stroke, malignancy, chronic obstructive pulmonary disease, alcohol abuse, diverticula of intestine, NSAIDs, systemic steroid, DMARDs, and monthly income.

† For AHR.

@ Rate: per 1,000 person-years.

**Table 3:** Independent predictors for GIP in all patients by Cox proportional hazard regression analyses.

**Variable**	**Crude HR^a^ (95% CI)**	**AHR^b^ (95% CI)**	***p*** **-value**
**Age (years)**			
<35	1 (reference)	1 (reference)	
35–45	3.49 (1.02–11.97)	3.57 (1.04–12.29)	0.044
45–55	2.37 (0.70–8.05)	2.36 (0.69–8.07)	0.172
55–65	3.96 (1.20–13.09)	3.67 (1.09–12.30)	0.035
65–75	7.70 (2.38–24.90)	6.48 (1.95–21.55)	0.002
75–85	14.24 (4.36–46.47)	10.70 (3.13–36.50)	<0.001
≥85	21.80 (4.87–97.69)	16.32 (3.50–76.06)	<0.001
**Sex**			
Female	1 (reference)	1 (reference)	
Male	1.47 (1.05–2.06)	1.28 (0.90–1.82)	0.174
**Underlying comorbidity**			
Diseases of the esophagus, stomach, and duodenum	1.19 (0.85–1.67)	0.78 (0.53–1.13)	0.191
Hypertension	2.12 (1.52–2.96)	1.13 (0.75–1.69)	0.568
Mental disorder	1.73 (1.22–2.45)	1.37 (0.94–2.01)	0.104
Liver disease	1.53 (1.05–2.23)	1.14 (0.74–1.73)	0.557
Hyperlipidemia	1.29 (0.85–1.95)	0.85 (0.54–1.36)	0.505
Renal disease	1.61 (1.05–2.47)	1.13 (0.72–1.79)	0.591
Coronary artery disease	1.61 (1.06–2.45)	0.78 (0.49–1.24)	0.287
Diabetes	1.78 (1.18–2.68)	1.20 (0.76–1.88)	0.438
Stroke	2.47 (1.45–4.23)	1.23 (0.70–2.18)	0.473
Malignancy	2.83 (1.63–4.93)	1.85 (1.05–3.27)	0.033
Chronic obstructive pulmonary disease	5.04 (3.03–8.39)	2.42 (1.39–4.21)	0.002
Alcohol abuse	3.51 (1.43–8.58)	2.68 (1.05–6.84)	0.040
Diverticula of the intestine	6.35 (2.02–19.94)	3.10 (0.96–10.00)	0.058
**Medications**			
NSAIDs	0.75 (0.38–1.51)	0.60 (0.29–1.22)	0.156
Systemic steroids	1.54 (0.85–2.80)	1.22 (0.67–2.23)	0.510
DMARDs	1.43 (0.78–2.61)	1.73 (0.94–3.19)	0.078
**Monthly income ($NTD)**			
<20,000	2.06 (0.91–4.69)	1.36 (0.58–3.18)	0.479
20,000–40,000	1.17 (0.45–3.01)	1.26 (0.49–3.25)	0.637
≥40,000	1 (reference)	1 (reference)	

GIP, gastrointestinal perforation; RA, rheumatoid arthritis; HR, hazard ratio; CI, confidence interval; NSAIDs, nonsteroidal anti-inflammatory drugs; DMARDs, disease-modifying antirheumatic drugs; NTD, New Taiwan Dollars.

Crude hazard ratio was adjusted for patients with and without RA and each variable.

Adjusted for patients with and without RA, age group, sex, diseases of the esophagus, stomach, and duodenum, hypertension, mental disorder, liver disease, hyperlipidemia, renal disease, coronary artery disease, diabetes, stroke, malignancy, chronic obstructive pulmonary disease, alcohol abuse, NSAIDs, systemic steroid, DMARDs, and monthly income.

## Discussion

This nationwide study demonstrated a trend of increased risk of GIP in patients with RA compared to patients without RA. Stratified analysis showed that the trend was more prominent for upper GIP. Female patients with RA had a significant increased risk of GIP compared to female patients without RA, but this was not observed in male population. Independent predictors for GIP were older age, malignancy, chronic obstructive pulmonary disease, and alcohol abuse.

The trend of increased risk of GIP in patients with RA may have multiple causes, including therapies and RA itself (7). Previous studies have revealed that NSAIDs, steroids, and DMARDs used in RA are responsible for GIP (7). This study showed that NSAIDs, systemic steroids, DMARDs were not independent predictors of GIP, which suggests RA itself may play more important role in the occurrence of GIP. This study showed that patients with RA had a trend of increased risk of upper GIP compared with patients without RA, which was compatible with previous studies (4–8, 14). The AHR of lower GIP in patients with RA was 1.24 compared with patients without RA; however, the difference was not significant (95% CI 0.73–2.09, *p* = 0.432). The possible reason for the non-significance is the relatively small sample size because of the low incidence rate of GIP.

This study shows that the increased risk of GIP was only found in female patients with RA, which is a new and interesting finding. Previous studies of intestinal perforation did not find significant difference between the two sexes (1, 15). A Chinese study also reported that spontaneous colonic perforation commonly occurred in patients aged >60 years and no difference was found between the two sexes (15). In this study, the incidence rates of GIP between male and female sexes among patients without RA were 1.1/1,000 and 0.5/1,000 person-years, respectively, which suggests a higher incidence in male patients in the non-RA population. However, the incidence rates GIP between male and female sexes in the RA population were 1.1/1,000 and 1.0/1,000 person-years, respectively, which suggests that the effect of RA on GIP was more prominent in female patients. The mechanism of the sex difference for GIP in RA remains unknown. A national data reported that depression, fibromyalgia, and hypothyroidism are more frequent in women than in men with RA (16), which suggests that the sex difference in this study is meaningful and needs further investigation.

In addition to RA, this study showed that older age, malignancy, chronic obstructive pulmonary disease, and alcohol abuse were independent predictors of GIP, which was compatible with previous studies (1). Older people have higher risks for previous abdominal surgeries and risk factors for ischemia, including smoking, coronary artery disease, and coagulation dysfunction, which could explain why older age is a predictor of GIP (1). Patients with chronic obstructive pulmonary disease use steroid for disease control, which may contribute to peptic ulcer disease and subsequent GIP (17). Alcohol and its metabolites can damage the gastrointestinal tract and liver by promoting intestinal inflammation through multiple pathways, including altering intestinal microbiota, increasing the permeability of the bowel mucosa, and affecting immune homeostasis (18).

As major strength, this study clarifies an unclear issue about the risk of GIP between patients with and without RA in an Asian population using a nationwide database. This study also found that RA has a more significant effect on the occurrence of GIP in female population than in the male population. The limitations are as the follows. First, the subtypes of RA (seropositive and seronegative) and detail medication dosage, including NSAIDs, systemic steroids, and DMARDs were not recorded in the database we used. Second, the number of developments of GIP was relatively small, which may contribute to the non-significant difference between patients with and without RA in the analyses. Further studies recruiting more patients and detail of the subtypes of RA (seropositive and seronegative) and medications, and investigation on the sex difference in the development of GIP in RA patients are warranted in the future.

## Conclusions

This nationwide population-based cohort study showed a trend of increased risk of GIP in patients with RA than in those with RA, especially true for upper GIP. Female patients with RA were more vulnerable to GIP than male patients with RA. However, further investigation is needed to understand the mechanism underlying the sex difference. More attention is needed in treating patients with RA, particularly in female patients, older patients, and those with malignancy, chronic obstructive pulmonary disease, and alcohol abuse.

## Data availability statement

The original contributions presented in the study are included in the article/supplementary material, further inquiries can be directed to the corresponding author/s.

## Ethics statement

The studies involving human participants were reviewed and approved by 10202-E12. Written informed consent for participation was not required for this study in accordance with the national legislation and the institutional requirements.

## Author contributions

T-CC and C-CHu designed and conceived this study and wrote the manuscript. C-HH and Y-CC performed the statistical analysis and wrote the manuscript. W-CK, K-CC, C-CC, C-CHs, H-TK, and H-JL provided professional suggestions and wrote the manuscript. All authors read and approved the final manuscript.

## Funding

This study was supported by the Grants Physician-Scientist 11001, CMHCR10954, CMFHR109101, CMFHR10991, and CMFHR111129 from the Chi Mei Medical Center.

## Conflict of interest

The authors declare that the research was conducted in the absence of any commercial or financial relationships that could be construed as a potential conflict of interest.

## Publisher's note

All claims expressed in this article are solely those of the authors and do not necessarily represent those of their affiliated organizations, or those of the publisher, the editors and the reviewers. Any product that may be evaluated in this article, or claim that may be made by its manufacturer, is not guaranteed or endorsed by the publisher.

## Abbreviations

AHR: adjusted hazard ratio
ATC: Anatomical Therapeutic Chemical
CI: confidence interval
DMARDs: disease-modifying antirheumatic drugs
GIP: gastrointestinal perforation
ICD-9-CM, International Classification of Diseases, Ninth Revision: Clinical Modification
NHIRD: National Health Insurance Research Database
NSAIDs: nonsteroidal anti-inflammatory drugs
NTD: New Taiwan Dollars
RA: rheumatoid arthritis.
